# Effects of Biochar Amendment and Nitrogen Fertilizer on RVA Profile and Rice Grain Quality Attributes

**DOI:** 10.3390/foods11050625

**Published:** 2022-02-22

**Authors:** Izhar Ali, Anas Iqbal, Saif Ullah, Ihsan Muhammad, Pengli Yuan, Quan Zhao, Mei Yang, Hua Zhang, Min Huang, He Liang, Minghua Gu, Ligeng Jiang

**Affiliations:** 1 College of Agriculture, Guangxi University, Nanning 530004, China; izharali48@gmail.com (I.A.); saif2012aup@gmail.com (S.U.); ihsanagrarian@yahoo.com (I.M.); pengliyuan@gxu.edu.cn (P.Y.); zq503730540@163.com (Q.Z.); zh347029559@163.com.com (H.Z.); lianghe@gxu.edu.cn (H.L.); gumh@gxu.edu.cn (M.G.); 2 College of Life Science and Technology, Guangxi University, Nanning 530004, China; anasiqbalagr@gmail.com; 3 College of Forestry, Guangxi University, Nanning 530004, China; fjyangmeri@126.com; 4 Crop and Environment Research Center, College of Agronomy, Hunan Agricultural University, Changsha 410128, China; mhuang@hunau.edu.cn

**Keywords:** RVA profile characteristics, rice milling and appearance quality, biochar, rice

## Abstract

Improving rice production in modern agriculture relies heavily on the overuse of chemical fertilizer, which adversely affects grain quality. Biochar (BC) application is well known for enhancing rice yield under reduced nitrogen (N) application. Therefore, we conducted a two-year field experiment in 2019 and 2020 to evaluate RVA profile characteristics, grain milling, and appearance qualities under four BC rates (0, 10, 20, 30 t ha^−1^) in combination with two N levels (135 and 180 kg ha^−1^). The results showed that BC at 30 t ha^−1^ along with 135 kg N ha^−1^ improved rapid visco-analyzer (RVA) profile attributes, including peak viscosity (4081.3), trough viscosity (3168.0), break down (913.3), final viscosity (5135.7), and set back (1967.7). Grain yield, grain rain length, milled rice rate, percent grains with chalkiness, amylose, and starch content were improved by 27%, 23%, 37%, 24%, 14%, and 8%, respectively, in the plots treated with the combination of 30 t BC ha^−1^ and 180 kg N ha^−1^. A positive coefficient of correlation was observed in RVA profile, milling, and apparent quality of rice with soil properties. These results suggested that BC at 20 to 30 t ha^−1^ in combination with 135 kg N ha^−1^ is a promising option for enhancing grain yield, RVA profile, appearance, and milling quality.

## 1. Introduction

Rice crop production and quality enhancement are a great challenge to overcoming the rising global population’s need [1]. The modern farming system has played a critical role in feeding the world’s population after green revaluation [2]; however, it is dependent on the overuse of chemical fertilizers [3]. The overuse of inorganic fertilizer has significantly reduced soil health, which is a major concern for agricultural output and grain quality. Furthermore, higher N fertilizer is not eco-friendly, i.e., increased ground water pollution, soil acidification, and boosts greenhouse gas emission [4]. In addition, continuous use of N fertilizer for a long time also showed significant reduction in soil microbial diversity and decreases in soil fertility, thus diminishing the growth and production of crops [5]. It has been reported that artificial fertilizer are usually applied in higher rates to crops than their requirement in China to obtain a higher output [1,6]. Farmers believe that using higher doses of chemical fertilizers is the easy, dependable way to increase crop production [7] while ignoring the awful impacts on soil, crop quality, and environment [8]. To cope with these problems, alternatively, BC amendment is well known for improving soil physiochemical properties [9] and crop physiology without increasing chemical fertilizer usage [3,10].

Biochar (BC) is an organic compound derived from biological organic material (biomass) through pyrolysis. BC addition to soil is considered a soil conditioner due to improving soil fertility and abating climate change by sequestering carbon [11]. Furthermore, BC application can increase crop growth by improving soil quality, which includes increasing beneficial microbial activity, improving water storage, and suppressing soil-borne disease; therefore, BC could be a useful soil amendment for crop production [3,9,11,12]. Applying BC in combination with N fertilizer improved rice starch metabolism enzymes and related gene expressions [13]. Moreover, BC coupled with N fertilizer also influences grain quality traits, i.e., amylose, starch, and protein contents of rice, compared to control plots [3,14].

Rapid visco-analyzer (RVA) profile of rice grain starch is a pasting curve generated from rice flour during a heating–high-temperature cooling process [15]. To know the viscosity properties of starch in rice, the RVA serves as a physical index for estimating the viscosity properties of rice starch, breakdown (BD), setback (SB), and peak viscosity (PV). These parameters are closely related to rice cooking and eating quality and could be useful tools for assessing rice grain quality [16,17]. It has been documented that rice grain RVA traits, including PV, BD, SB, and consistency (CSV), were affected by different environmental conditions [18], exogenously applied plant growth regulators [18], and N application [19,20].

Nitrogen (N) fertilizer plays a significant role in rice grain appearance quality, starch content, amylose content, and RVA traits, a significant and non-negligible environmental component [15,16,21]. Previously, it was shown that N fertilizer does not promote rice quality; however, many research findings have shown that the right amount of nitrogen can maintain and improve rice quality. Likewise, Sing et al. [22] reported that lower amylose content and higher pasting temperature, gelatinization-transition temperatures, and gelatinization enthalpy were recorded in rice with N application. Furthermore, improved contents of protein, amylose, and alkali spreading value were recorded in the moderate application of N fertilizer [21]. According to Gu et al. [23], starch PV, hot paste viscosity (HPV), cool paste viscosity (CPV), and BD decreased with increasing the N fertilizer levels, consequently deteriorating the eating quality. An increase in N application rate might increase rice grain protein and component protein; however, inappropriate application of N will diminish yield and quality. Furthermore, N application in combination with BC might improve seed amylose content and protein content [3,16].

Rice crop is one of the major food staples worldwide, while its noodle is considered a traditional food of southern China. China consumes almost 2.2 times the global average in rice with a per capita consumption of 100 to 120 kg year^−1^ [24,25]. Rice grain quality traits include nutritional values, physical appearance, milling, and cooking and eating qualities [18]. In China, some rice verities have more yield and better amylose content but poor cooking and eating quality, which are used to produce rice noodles [18]. Brown rice percentage, milled rice percentage, and head rice percentage are common milling quality assessment criteria that reflect the proportion of whole kernels (head rice or head milled rice) and broken kernels produced during rough rice milling [18]. Although numerous studies have been conducted on amylose content and RVA profile of rice grain nutritional quality [15,18,22,23,26], to our knowledge, this is the first experimental study to evaluate the responses of noodle rice grain RVA profile characteristics and other grain quality attributes to different BC and N applications under paddy field conditions.

Despite the importance of rice RVA profile characteristics, nutritional values, physical appearance, milling, and cooking performances to world food security and the economy, a lack of studies on these attributes leaves a significant gap in the literature on this issue. In the current study, we used various combinations of BC and N fertilizers to fill this gap to find these attribute responses. Therefore, the objectives of this study were to evaluate the impact of BC in combination with N fertilizers on rice grain milling quality, grain appearance quality, grain viscosity traits, and their relationship to soil physiochemical properties under field conditions.

## 2. Material and Methods

### 2.1. Location, Test Time, and Materials

**Figure 1:**
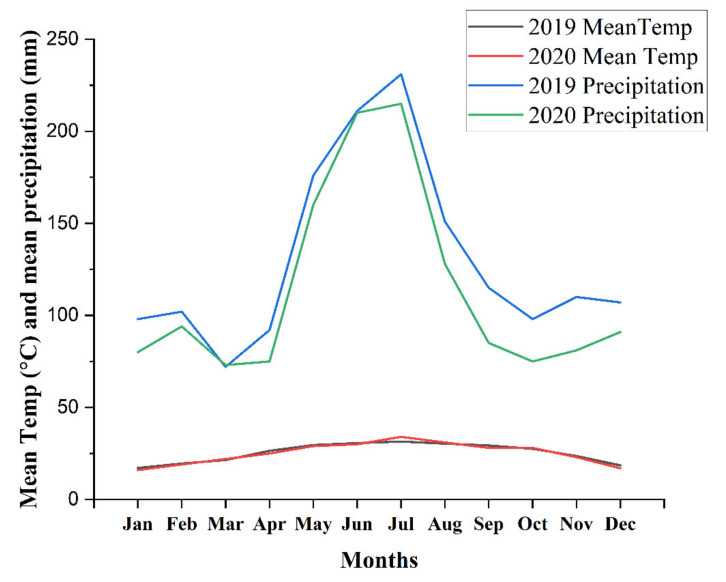
Mean temperature and mean precipitation of the experimental site during both years.

A two-year field experiment was conducted in Nanning, Guangxi, with different BC and N fertilizer treatments in 2019 and 2020. The climate onsite is subtropical monsoon, and the data on mean temperature and mean precipitation are represented in Figure 1. The soil (0–20 cm) is graded as Ultisols and is slightly acidic (pH 5.94), soil organic carbon (SOC) 15.10 g kg^−1^, soil organic matter 25.8 g kg^−1^, total N (TN) 1.35 g kg^−1^, available N (AN) 134.7 mg kg^−1^, available phosphorous (23.1 mg kg^−1^), and available potassium (AK 233.6 mg kg^−1^, with 1.36 g cm^−3^ soil bulk density (BD).

### 2.2. Biochar Production

Cassava straw was used in kilns with the temperature ranging 300 °C to 500 °C, following the method previously documented by Ali et al. [3]. The properties of BC were C (674.00 g·kg^−1^), H (3.81 g·kg^−1^), P (46.33 g·kg^−1^), N (5.43 g·kg^−1^), K (48.33 g·kg^−1^), and S (2.39 g·kg^−1^); specific area (2.46 m^2^ g^−1^); and pore diameter (3.37 nm) with C:N ratio (124.12) and are presented in our previous study. Table 3 in [3]).

### 2.3. Experimental Design

The field experiment was conducted in a randomized complete block (RCB) design with three replications and a plot size of 3.9 m × 6 m (23 m^−2^) during 2019 and 2020. Four different rates of BC (0, 10, 20, and 30 ton ha^−1^ equal to 0, 23.4, 24.8, and 70.2 kg plot^−1^, respectively) and two levels of N (135 and 180 kg ha^−1^) were applied. The groups of treatment were as follows: (i) N1B0- N135 kg ha^1^ + 0 BC t ha^−1^; (ii) N1B1- N135 kg ha^1^ + 10 BC t ha^−1^; (iii) N1B2- N135 kg ha^1^ + 20 BC t ha^−1^; (iv) N1B3- N135 kg ha^1^ + 30 BC t ha^−1^; (v) N2B0- N180 kg ha^1^ + 0 BC t ha^−1^; (vi) N2B1- N180 kg ha^1^ + 10 BC t ha^−1^; (vii) N2B2- N180 kg ha^1^ + 20 BC t ha^−1^; and (viii) N2B3- N180 kg ha^1^ + 30 BC t ha^−1^. The cultivar “Zhenguiai” of noodle rice was utilized as a model crop. The plastic trays were arranged for the nursery, and uniform seedlings (after 25 days) were transplanted as 13 rows per plot and two seedlings per hill. The locally recommended doses of phosphorus and potassium were applied at the rate of 75 kg ha^−1^ and 150 kg ha^−1^, respectively. The BC was introduced once to the field 25 days before the transplantation of seedlings in 2019..

### 2.4. Grain Yield, Quality and RVA Profiles Assessment

Rice was harvested at the physiological maturity stage from each treatment and was stored for 3 months at 12% moisture content. Grain yield is expressed as kg ha^−1^. After 3 months, three kilograms of rice were randomly selected from each plot and milled with a small Miller (Kett, Pearlest, Tokyo, Japan). A super-high-speed mill grinds the material into powder, which is then filtered via a 100-mesh screen. Subsequently, the amylose and starch content were assessed by using the procedure described by Juliano et al. [27] and Zhu et al. [28].

To determine the rice RVA profile, another randomly selected three kilograms of rice were sent to Hunan Agricultural University’s Crop Multi-cropping Planting Model and Agronomic Technology Innovation Platform (Super-4 Rapid Visco-Analyzer, Newport Scientific Instruments, Sydney, Australia). Each sample was analyzed in triplicate. The following parameters were determined: peak viscosity, trough viscosity, break down, final viscosity, set back, reply value, peak time and pasting temperature, strip breaking rate (%), expansion rate (%), uniformity (%), the value of dissolving starch, and evenness.

For grain quality determination, a total of 100 grains were scanned in order to assess the appearance quality as previously described by Fahad et al. [18] (Epson Expression 1680 Professional, Epson, Los Alamitos, CA, USA). Digital photographs of each head rice subsample were generated using a scanner with a black background to increase the contrast between the chalky and translucent portions in the images. The image analysis software (Image J, the National Institutes of Health, Washington, MD, USA) was used to examine the attributes in the digital images, including grain length, grain width, chalky grain percentage, and chalkiness.

### 2.5. Statistical Analysis

Statistix software, version 8.1 (Analytical Software, Tallahassee, FL, USA) was used to analyze the data by using factorial ANOVA. The least significant difference test was used to separate the differences between treatments at the 0.05 probability level. R software (corrplot package) was used to perform correlation analysis for all measured traits.

## 3. Results

### 3.1. Soil Physiochemical Properties

Data on soil physiochemical properties as influenced by different BC and N fertilizer amendments are shown in our recent published article (Table 2 in [12]). The results showed that BC in combination with N fertilizer more notably improved pH, available potassium (AK), available phosphorus (AP), total nitrogen (TN), soil organic carbon (SOC), and bulk density (BD) than did solely N treatments. The higher values of pH (7.13), TN (1.96 g kg^−1^), AP (31.59 mg kg^−1^), AK (277.71 mg kg^−1^), and SOC (17.12 g kg^−1^) resulted in higher BC-treated soil along with 180 kg N ha^−1^. Soil BD (1.24 g m^−3^) was decreased with an increase in BC amendment under both N rates. The lowest values of soil physiochemical properties except BD were recorded in solely N-treated plots.

### 3.2. Grain Yield

**Figure 2:**
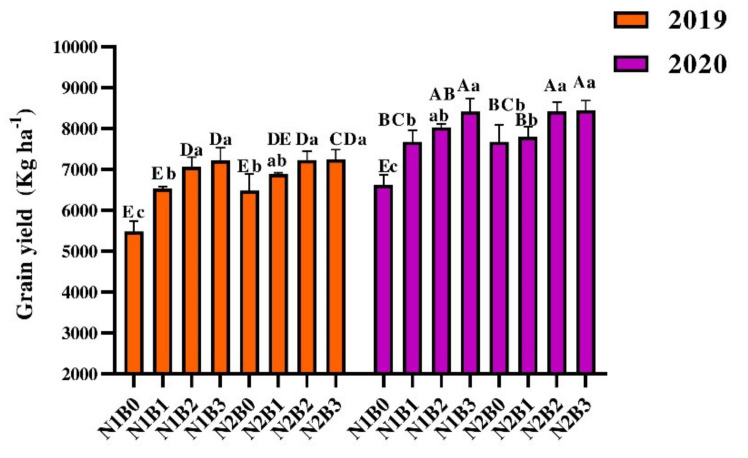
Effect of biochar and nitrogen fertilizer on grain yield. Bars represent the standard errors of the means. Different small and capital letters show significant differences between the treatments within the same year and between the years in the same treatment, respectively, via LSD test at *p* ≤ 0.05. N1 and N2 represent 135 and 180 kg ha^−1^ nitrogen; B0, B1, B2, and B3 represent 0-, 10-, 20-, and 30-ton biochar ha^−1^.

An increase in grain yield was recorded in BC applied treatments as compared to sole N treatments. Grain yield was significantly higher in 2020 as compared to 2019 (Figure 2). Among the treatments in 2019, N1B2, N1B3, N2B2, and N2B3 ameliorated grain yield by 25% to 30% as compared to solely N treatments. Similarly, during 2020, grain was greater by 32% in 30 ton BC ha^−1^ along with 135 kg N ha^−1^ compared to solely N treatments. Among the years, the grain yield was improved by 16% in 2020 as compared to 2019. The lowest grain yields of rice during 2019 (5481.48 kg ha^−1^) and in 2020 (6621.08 kg ha^−1^) were recorded in non-BC applied treatments.

### 3.3. Grain RVA Profile Properties

Data on rapid visco-analyzer (RVA) as influenced by BC and N fertilizer are presented in Table 1. These grains attributes responded differently to BC and N fertilizers. BC application significantly alters peak viscosity, trough viscosity, break down, and reply value as compared to non-BC-treated soil across N application. The BC amendment rate (30 t ha^−1^) in combination with higher N level (180 kg ha^−1^) as compared to the other treatments affected these grain traits, not including the values of setback and peak time across the BC amendments. Among the treatments in 2019, higher values of peak viscosity (4081.3), trough viscosity (3168.0), break down (913.3), final viscosity (5135.7), and set back (1967.7) were recorded in higher BC amendments. The lowest values of rice grain peak viscosity (3851.3) and trough viscosity (3070) and final viscosity (4919.0) were recorded in the plots treated with no BC plus 180 kg N ha^−1^, whereas lower breakdown and setback values were recorded by 632.3 and 1827.3 in 20 t B ha^−1^ + 135 kg N ha^−1^ and 0 t B ha^−1^ + 135 kg N ha^−1^, respectively. Reply value, peak time, and pasting temperature were reduced with increasing BC and N fertilizer. However, no statistical dissimilarities were observed for reply value, peak time, and pasting temperature among the treatments. However, the highest values of 1134, 6.18, and 82.6 for reply value, peak time, and pasting temperature were recorded in the control treatment (0 t B ha^−1^ + 135 kg N ha^−1^). In general, our results showed that BC along with N fertilizer significantly altered grain rapid visco-analyzer data of noodle rice. Compared to non BC treatments in 2020, peak, trough, and final viscosity, set back, and reply value were higher by 2.64%, 3.30%, 3%, 43.59% and 1.18% in the treatments of 30 t BC combined with 180 kg N ha^−1^.

### 3.4. Rice Grain Appearance and Milling Qualities Properties

Rice grain appearance and milling quality, including grain area, grain width, grain length, head rice, milled rice rate, percent grains with chalkiness, and percent area of chalkiness endosperm, as influenced by combined application of BC and N fertilizer, were determined by using Epson Expression 1680 Professional Scanner, Epson, America (Table 2). Results showed that grain area, grain width, and the percent area of chalkiness endosperm were not significant (*p* > 0.05) for BC, N, and their combined application during both years (2019 and 2020). Across N rates, head rice, milled rice rate, and percent grains with chalkiness were not significantly affected by BC amendment rates. However, N application significantly enhanced head rice, milled rice rate, and percent grains with chalkiness by 2%, 10.63%, and 3.03%, respectively, in the higher N rate as compared to the lower N rate. Across N rates, BC amendment at the rate of 30 t ha^−1^ resulted in lengthier grain by 15% as compared to non-BC treatments. The interaction of B × N showed that the grain lengths of rice were significantly higher by 23% in the treatment had 30 t BC ha^−1^ along with 135 kg N ha^−1^. Similarly, milled rice rate and percent grains with chalkiness were higher by 37% and 24% in 30 t BC ha^−1^ along with 180 kg N ha^−1^-treated plots.

### 3.5. Amylose and Starch Content Properties

**Figure 3:**
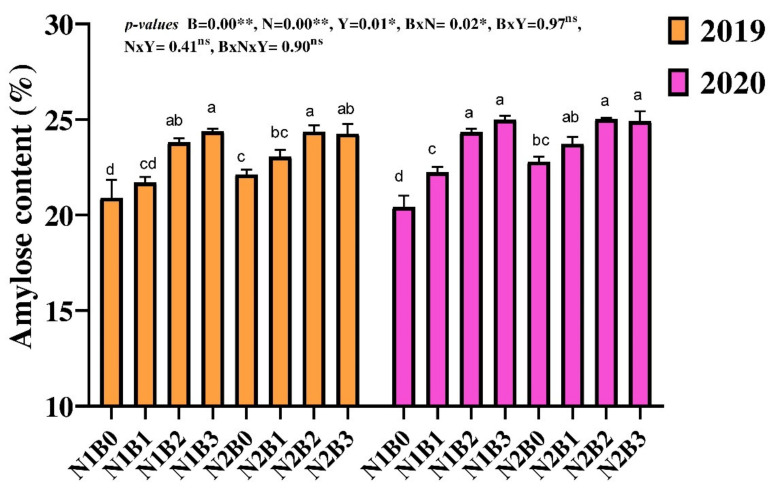
Changes in amylose content of rice in response to different biochar and nitrogen fertilizer. Different letters above the column are significantly different from each paper. Bars represent the standard errors of the means. ** and * on the B, N, and Y indicate significance at the 0.01 and 0.05 probability levels, respectively. ns: non-significant. Columns with different letters are significantly different at *p* < 0.05. N1 and N2 represent 135 and 180 kg ha^−1^ nitrogen; B0, B1, B2, and B3 represent 0-, 10-, 20-, and 30-ton biochar ha^−1^.

Data regarding amylose content showed that BC addition at the rate of 20 to 30 t ha^−1^ increased 14% amylose as compared to non-BC treatments across N applications (Figure 3), whereas among N levels, plots treated with 180 kg N ha^−1^ improved amylose content by 3% as compared to 135 kg N ha^−1^-treated plots. The interaction of B × N did not show a significant effect on amylose content; however, the combination of 20 to 30 t BC ha^−1^ with both low- and high-N application improved amylose content as compared to solely N-applied plots. The overall results showed that the lowest values of all these traits were recorded in non-BC-treated plots under both N rates.

**Figure 4:**
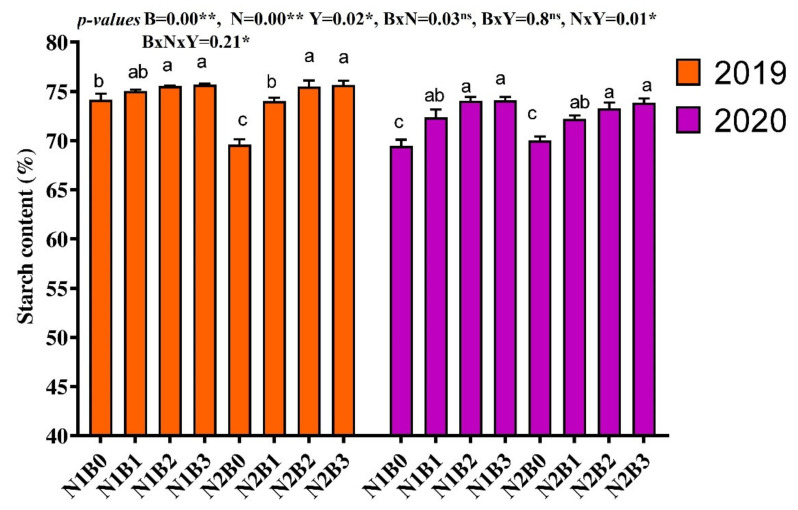
Changes in starch content of rice in response to different biochar and nitrogen fertilizer. Bars represent the standard errors of the means. ** and * on the B, N, and Y indicate significance at the 0.01 and 0.05 probability levels, respectively. ns; non-significant. Columns with different letters are significantly different at *p* < 0.05. N1 and N2 represent 135 and 180 kg ha^−1^ nitrogen; B0, B1, B2, and B3 represent 0-, 10-, 20-, and 30-ton biochar ha^−1^.

Starch content was significantly affected by BC, N, and their combined application (Figure 4). Results showed that among the treatments, BC at the rate of 20 to 30 t ha^−1^ in combination with both low (135 kg N ha^−1^) and high (180 kg N ha^−1^) N significantly increased starch content by 8.47% and 6.67% as compared to solely N applied treatments. Furthermore, across BC applications, 135 kg N ha^−1^ improved starch content by 2% as compared to 180 kg N ha^−1^, whereas across N treatments, BC application at the rate of 20 and 30 t ha^−1^ enhanced starch content by 5.2% and 6.01% as compared to non-BC treatment. However, there was no statistical difference recorded among 20 and 30 t BC ha^−1^ for the starch content of rice.

### 3.6. Relationship of Soil Properties, Rice Grain Milling, Appearance Qualities, and RVA Profile

**Figure 5:**
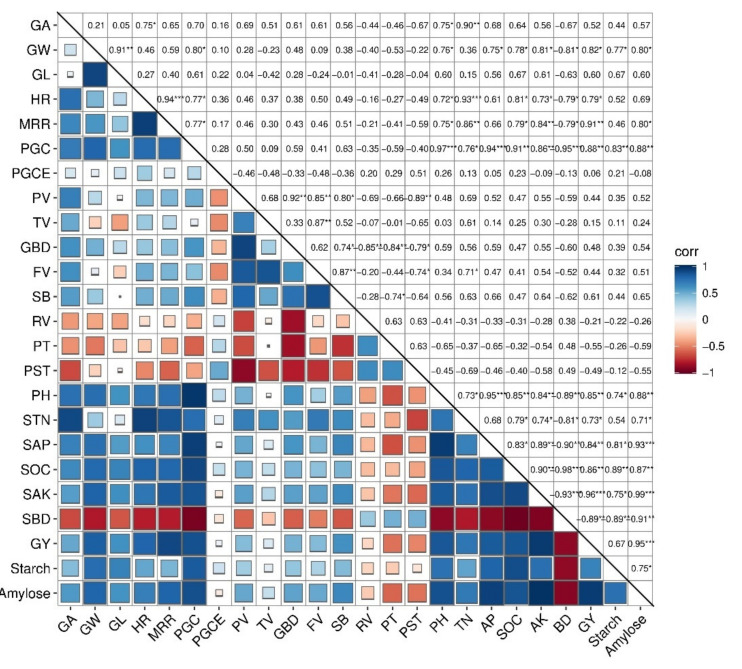
Correlations heat map of soil properties, grain appearance quality, milling quality, and RVA profile of rice using R software. Square with red to dark blue color indicates the relationship from negative to positive. Square size from small to big indicates the moderate positive/negative to strongly negative/positive relationship. The values in the figure showed the R-squared values. AP, available phosphorus; SOC, soil organic carbon; TN, total nitrogen; AK, available potassium; BD, bulk density; PV, peak viscosity; TV, trough viscosity; BD, break down; FV, final viscosity; SB, set back; RV, reply value; PT, peak-time; PST, pasting-temperature; GA, grain area; GW, grain width; GL, grain length; HR, head rice; MRR, milled rice rate; PGCE, percent area of chalkiness endosperm (%); PGC, percent grains with chalkiness; GY, grain yield. *, ** and *** show statistical significance at *p*>0.05, *p*>0.01 and *p*>0.001, respectively.

The correlation analysis showed that soil pH, TN, AP, SOC, and AK strongly positively correlated with grain yield, grain width, grain length, head rice, milled rice, peak viscosity, trough viscosity, break down, and set back (Figure 5), whereas soil BD showed a negative relationship to grain width, grain length, head rice, milled rice, peak viscosity, trough viscosity, break down, and set back. Similarly, pasting temperature (R^2^ = −0.85), peak time (R^2^ = −0.84), and reply value (R^2^ = −0.79) were found to have a strongly negative association with seed break down. Soil pH (R^2^ = 0.76 and R^2^ = 0.60), TN (R^2^ = 0.36 and R^2^ = 0.15), AP (R^2^ = 0.75 and R^2^ = 0.56), SOC (R^2^ = 0.78 and R^2^ = 0.67), and AK (R^2^ = 0.81 and R^2^ = 0.61) were strongly, positively correlated with grain width and grain length, respectively. Furthermore, grain yield, starch content, and amylose content had a significantly positive correlation with soil properties (pH, SOC, AP, TN, and AK). Similarly, starch and amylose content were also positively correlated with PV, TV (R^2^ = 0.35, R^2^ = 0.52), FV (R^2^ = 0.11, R^2^ = 0.24), BD (R^2^ = 0.39, R^2^ = 0.54), and SB (R^2^ = 0.44, R^2^ = 0.65) while negative correlated with RV (R^2^ = −0.22, R^2^ = −0.26), PT (R^2^ = −0.26, R^2^ = −0.59), and PST (R^2^ = −0.12, R^2^ = −0.55). Milled rice rate and head rice percent grains with chalkiness were also strongly, positively correlated with soil pH, SOC, AP, TN, and AK. Reply value, peak time, and pasting temperature were recorded to have a negative or no relationship with soil pH, TN, AK, AP, and SOC.

## 4. Discussion

Rice grain appearance and milling quality are essential for the people whose staple food is rice grain or its noodle [15]. Rice grain quality can be influenced by different environmental conditions and cultivars [15,18]. Likewise, rice grain quality is adversely affected by the overuse of chemical fertilizers, which is currently used for improving grain yield in modern agriculture [21]. Nowadays, research on improving grain yield and quality without disturbing soil quality is considered novel research. Therefore, in the present study, the impacts of different BC and N applications on rice grain milling and appearance qualities and grain RVA profile characteristics were assumed. In the present study, soil physiochemical properties were improved with the combined application of BC and N fertilizer. Increasing BC at 30 t ha^−1^ under both low- (135 kg ha^−1^) and high-N (180 kg^−1^) levels improved soil properties. The possible reason for these improvements is the structure of and chemical composition of BC [3]. BC had a high-pH level in our study, and when applied to acidic soil, it improved soil pH and other soil properties. Our findings are in line with De-Sousa-Lima et al. [29], Ullah et al. [9], and Ali et al. [3], who reported that the addition of BC to soil improved soil pH, SOC TN, AK, and AP under paddy field conditions. Soil treated with BC amendment enhances the activities of soil enzymes and microbial biomass, which consequently improves soil physicochemical properties [30,31,32].

Rice grain yield was increased with increasing BC rate combined with both N (low and high) applications. Solely N-treated plots produced the lowest grain yield during both years. The possible reason for these increments might be due to the chemical properties of BC applied to soil, which improved the soil physiochemical properties and thus improved grain yield [12]. The positive relationship of soil properties with grain yield is confirmed in our study (Figure 5). Another possible explanation is that BC in combination with N application improves N uptake in rice [14]. Improving N uptake in plants consequently increases photosynthetic attributes and N metabolism enzymes in rice [14,21,33], thus improving grain yield. The variation among these two years might be due to BC being a slow-released fertilizer [34], which can contribute to soil up to several years because BC had long-term effects on soil [35]. Therefore, we assumed that the grain yield of 2020 was increased due to the slow release of BC, which contributed more in the second year to soil fertility. Similar results of rice yield in BC-applied soil were reported by Ullah et al. [9] and Fahad et al. [18]. Grain viscosity traits, including peak, trough, and final viscosity, were increased with higher N rates in the current study. However, moderate values of these attributes were obtained in the high-BC treatments in combination with a lower N rate. Set back and peak time were not affected by both BC and N fertilizer and their combined application. The final and peak viscosity difference was attributed to set back and is believed to be a grain starch quality parameter. Changes in the RVA profile of noodle rice grain in BC-treated plots were ascribed to the enhancement in physiochemical properties of soil [13]. Combine application of BC and N fertilizer can influence soil pH, SOC, AK, TN, and AP due to BC, which has nutrient content [3]. In addition, BC-enhanced soil pH in acidic soil, which has improved soil fertility compared to non-BC-treated soil [36,37]. Zhao et al. [38] reported that BC application improved soil mineral content by preservation, and this enhanced N availability, which can improve N uptake by rice plant [13,39,40], consequently increasing rice grain quality [3]. Further improvement in RVA characteristics in the current study was confirmed by the correlation analysis in our study that RVA profile characteristics were positively correlated with soil properties (Figure 5).

Rice grain quality can be unexpectedly influenced by soil fertility and environmental temperature during rice kernel development [15,16,41]. For example, N application affects kernel development and considerably reduces head rice yield [42]. Similarly, increased chalkiness formation [43,44], reduced amylose content [21,45], and the resulting smaller grain size [5] were observed in the higher N application. Moreover, N fertilizer combined with BC amendment positively affected rice grain quality in the paddy fields [3,13]. In the present study, BC combined at the rate of 20 to 30 t ha^−1^ improved grain milling, appearance quality, and amylose content as compared to solely N fertilizer treatments. Increments in these traits attributed to the improvement in soil physiochemical properties, soil enzymatic activities, and nutrients uptake of plants [12]. Furthermore, in combination with N fertilizer, BC also improved rice root morphological attributes, which consequently improved the growth and grain N content of rice [3,9,12,45,46]. Previously, it was reported that rice grain quality (cooking and sensory qualities) was closely linked to grain rheological properties, influenced by starch accumulation and deposition [18,40,47]. Furthermore, combined BC and N fertilizer also improved starch content, starch metabolism enzymes, and the related gene expression of rice [13,48]. Similar results were reported by Ullah et al. [9], who conducted a pot experiment and stated that amylose content and other grain qualitative traits were improved in the combined application of BC and N fertilizer. The overall results showed that BC amendment combined with N can significantly mitigate the effect of overuse N application and its ill effects on rice grain appearance, milling, and quality aspects.

Correlation analysis showed a positive correlation among soil properties, grain yield, grain width and length, head and milled rice, peak viscosity, trough viscosity, break down, and set back. Furthermore, positive relationship among starch and amylose content with soil properties and viscosity traits were also obtained. Similar findings were reported by Xuan et al. [15] that amylose content was strongly positively correlated with FV, TV, SB, and grain BD. Shiji et al. [26] documented that RVA had a significant relationship with amylose content, in which amylose might affect the RVA traits to alter the taste value of rice. However, in contrast, a negative correlation of PV and BD with amylose content was recorded in 71 japonica and 68 Indica rice verities [49]. Furthermore, under a high-temperature environment, a significant correlation between amylose content and RVA profile during the grain filling stage was also observed [50]. In combination with N fertilizer, BC application improved amylose and starch content, which indirectly influences the noodle rice RVA profile.

## 5. Conclusions

Based on our results, we observed that grain yield, grain appearance, milling, and RVA profile traits were considerably improved in the combined application of BC and N fertilizer. The improvements in grain appearance and milling qualities, viscosity attributes, amylose, and starch contents of noodle rice were due to an improvements in soil physiochemical properties under BC application. Moreover, according to correlation analysis, appearance quality and viscosity characteristics of rice grain and grain yield are strongly dependent on soil pH, SOC, AK, TN, and AP. BC at the rate of 20 to 30 t ha^−1^ in combination with 135 kg ha^−1^ was the most effective treatment for improving rice grain yield and quality to overcome the rising global population’s need.

## Figures and Tables

**Table 1:** Impacts of biochar and nitrogen fertilizer on Rapid visco-analyzer (RVA) profile characteristics of rice.

BC	N	Peak Viscosity	Trough Viscosity	Break Down	Final Viscosity	Set Back	Reply Value	Peak Time	Pasting Temp
(ton ha^−1^)	(kg ha^−1^)								
2019									
0	135	3822.7 bc	3130.0 a	692.7 ab	4957.3 ab	1827.3 b	1134.7 a	6.18 a	82.6 ab
10	135	3802.0 bc	3114.7 a	687.3 b	4975.0 b	1860.3 ab	1173.0 a	6.13 a	81.8 bc
20	135	3717.7 bc	3085.3 a	632.3 b	4911.3 ab	1826.0 b	1193.7 a	6.20 a	83.4 a
30	135	3878.3 bc	3167.0 a	711.3 ab	5077.7 b	1910.7 a	1199.3 a	6.15 a	82.1 ab
0	180	3851.3 c	3070.0 a	781.3 ab	4919.0 ab	1849.0 b	1067.7 a	6.11 a	81.8 bc
10	180	3972.7 ab	3206.3 a	766.3 ab	5065.0 ab	1858.7 ab	1092.3 a	6.18 a	80.8 c
20	180	3848.3 bc	3162.3 a	686.0 b	5045.0 ab	1882.7 ab	1196.7 a	6.16 a	81.8 bc
30	180	4081.3 a	3168.0 a	913.3 a	5135.7 a	1967.7 a	1054.3 a	6.07 a	80.8 c
2020									
0	135	3699.7 c	2999.3 c	627.7 c	4901.7 b	1902.3 a	1202.0 a	5.97 b	81.4 a
10	135	3707.3 c	3021.3 ab	680.7 c	4862.0 b	1840.7 bc	1154.7 ab	6.01 a	82.2 a
20	135	3790.7 b	3103.0 a	699.3 c	5034.0 a	1931.0 a	1243.3 a	5.97 a	80.9 a
30	135	3873.0 a	3006.0 bc	769.3 b	4832.0 b	1826.0 bc	959.0 b	5.91 a	80.7 a
0	180	3717.7 c	3050.7 ab	675.3 c	4854.0 b	1803.3 c	1136.3 ab	5.93 a	80.6 a
10	180	3755.0 b	3142.3 a	674.0 c	5050.7 a	1908.3 a	1295.7 a	5.99 a	79.7 a
20	180	3797.3 b	3098.3 ab	754.3 b	4965.0 b	1866.7 b	1167.7 ab	5.96 a	80.6 a
30	180	3897.0 a	3124.0 a	901.3 a	5048.7 a	1924.7 a	1151.7 ab	5.89 ab	79.6 a
SOV									
BC		**	ns	*	ns	ns	**	**	*
N		**	*	ns	*	ns	ns	ns	**
Y(years)		**	*	ns	*	ns	ns	*	**
B × N		ns	ns	ns	**	*	ns	ns	**
B × Y		ns	ns	ns	ns	ns	ns	ns	ns
N × Y		ns	ns	ns	ns	ns	ns	ns	ns
N × B × Y		ns	ns	ns	ns	ns	ns	ns	ns

Note: ** and * show difference at 1% and 5%, respectively. Various letters (a, b, c,…) within the column show significant difference at 0.05 probability level according to least significant difference (LSD). SOV, source of variation; LSD, least significant differences; ns, non-significant; BC, biochar; N, nitrogen.

**Table 2:** The effects of biochar in combination with nitrogen fertilizer on the milling and appearance of grain qualities of rice.

2019 BC	N	Grain Area (mm^2^)	Grain Width (mm)	Grain Length (mm)	Head Rice (%)	Milled Rice Rate (100%)	Percent Grains with Chalkiness (%)	Percent Area of Chalkiness Endosperm (%)
0	135	8.78 a	2.24 a	2.13 c	85.04 b	53.07 b	36.52 b	11.35 ab
10	135	8.50 a	2.76 a	2.35 b	86.64 ab	64.85 ab	40.54 ab	14.03 a
20	135	8.50 a	2.88 a	2.32 bc	86.04 ab	64.11 ab	40.94 b	9.89 b
30	135	8.96 a	3.28 a	2.63 a	86.34 ab	68.19 a	43.87 ab	11.93 ab
0	180	8.49 a	2.47 a	2.15 c	85.97 ab	65.28 ab	37.25 b	10.79 ab
10	180	9.33 a	2.65 a	2.30 bc	87.02 ab	70.33 a	39.90 ab	10.81 ab
20	180	9.91 a	2.70 a	2.26 bc	87.47 a	72.93 a	44.21 ab	12.97 ab
30	180	9.91 a	2.96 a	2.30 bc	87.30 a	71.47 a	45.55 a	11.42 ab
2020								
0	135	9.04 a	2.49 b	2.25 c	83.57 b	52.4 c	37.42 b	8.68 a
10	135	8.77 a	2.8 ab	2.46 b	85.17 ab	67.52 ab	41.11 ab	9.69 a
20	135	8.76 a	3.35 a	2.43 bc	84.57 ab	63.44 b	41.84 ab	8.56 a
30	135	9.23 a	3.24 a	2.75 a	84.87 ab	73.19 ab	44.77 a	9.93 a
0	180	8.75 a	2.3 c	2.27 bc	83.84 b	70.28 ab	38.15 b	10.46 a
10	180	9.6 a	2.85 ab	2.41 bc	85.55 a	75.33 a	40.8 ab	9.15 a
20	180	10.18 a	3.09 a	2.38 bc	86.01 a	77.93 a	44.78 a	10.64 a
30	180	10.18 a	2.9 ab	2.41 bc	85.93 a	76.47 a	44.78 a	9.76 a
SOV								
BC		ns	*	**	**	**	**	**
N		*	ns	**	**	**	ns	ns
Y (years)		ns	ns	**	**	ns	ns	ns
B × N		ns	ns	**	ns	ns	ns	ns
B × Y		ns	ns	ns	ns	ns	ns	ns
N × Y		ns	ns	ns	ns	ns	ns	ns
B × N × Y		ns	ns	*	ns	ns	ns	ns

Note: ** and * show difference at the 1% and 5%, respectively. Various letters (a, b, c,...) within the column show significant difference at 0.05 probability level according to least significant difference (LSD). SOV, source of variation; LSD, least significant differences; ns, non-significant; BC, biochar; N, nitrogen.

## Data Availability

The data presented in this study are available on request from the corresponding author.
